# Bridging and bonding: The roles of brokerage and closure in mobilizing support provision in online support groups

**DOI:** 10.1371/journal.pone.0325108

**Published:** 2025-06-10

**Authors:** Sanguk Lee, Tai-Quan Peng

**Affiliations:** 1 Department of Communication Studies, Texas Christian University, Fort Worth, Texas, United States of America; 2 Department of Communication, Michigan State University, East Lansing, Michigan, United States of America.; University of Exeter, UNITED KINGDOM OF GREAT BRITAIN AND NORTHERN IRELAND

## Abstract

Social capital theory suggests that network structure influences human behavior. Based on this premise, this study investigates how two distinct network structures—brokerage and closure—affect support provision in Online Support Groups (OSGs). Twelve years of data were collected from an online cancer community based in South Korea. Using computational methods, we measured support behaviors, network structures, and social capital. The findings reveal that brokers, through exposure to non-redundant information, provide social support in larger volumes, with higher quality, and in a more timely manner. While closure has limited explanatory power for overall support behaviors, it specifically facilitates provision of improved quality of emotional support through one’s enhanced trust. Additionally, the results highlight the importance of recognizing the dynamic developmental stages of OSGs to fully understand the social mechanisms driving support provision. These findings offer significant insights into the mobilization of social support through network structures in the OSG context.

## 1. Introduction

Online support groups (OSGs) provide virtual platforms for individuals with various health conditions to exchange information and build connections. These digital communities empower patients and informal caregivers to supplement insufficient offline support [1], share practical advice, and access emotional support, which is often lacking in conventional medical channels. Since most OSGs rely on volunteers, the continued involvement of members is essential for the longevity of these groups. Since receiving and exchanging support are primary motivations for individuals’ participation in OSGs [2], providing timely and adequate social support plays a crucial role in ensuring the sustainability of these communities. Therefore, understanding the factors and mechanisms that drive and motivate individuals to provide social support is of both theoretical and practical importance for OSGs.

Social support provision is inherently social. While it is an individual behavior, the source, motivation, and driving force behind the behavior are deeply rooted in the social fabric. An individual’s social context, shaped by social structures—the organized patterns of relationships and positions that define how individuals interact within a community—plays a pivotal role in shaping, redirecting, and limiting social actions [3]. Despite previous studies highlighting the association between social network structure and reception of social support [4–6], research exploring the connection between network structure and support provision has primarily focused on knowledge contribution [7], leaving room for further investigation into social mechanisms within the broader landscape of social support behaviors and outcomes in the health context [8].

Drawing on the market metaphor, social capital theory offers a compelling framework for explaining the impact of resources derived from social networks on human behaviors. In this study, social capital theory serves as a theoretical lens to explore how specific social network structures, fostered through interactions and support exchanges in OSGs, facilitate the provision of social support. Specifically, this study examines two key structural concepts from social capital theory—brokerage (bridging disconnected groups) and closure (tight-knit connections)—to understand how these distinct network configurations generate diverse forms of social capital, such as access to non-redundant information and trust. Furthermore, it investigates how these different forms of social capital play varying roles in enabling the provision of different dimensions of social support.

The exploration of the relationship between social structure and social support behavior represents a promising research area. However, it should be noted that only a limited number of studies have pursued this trajectory [9]. Presumably, methodological constraints, such as the high cost associated with collecting extensive social network data, have hindered further investigation in this domain. Additionally, the conceptual challenges of defining and measuring various dimensions of social and individual metrics from behavioral data have further restricted progress in this line of inquiry. To overcome these limitations, we utilized computational methods and capitalized on the self-initiated nature of digital footprints collected from a popular Korean OSG. This approach provided us with a more reliable and valid means of measuring a large-scale support exchange network, social capital, and support provision. By surpassing previous methodological and conceptual barriers, our study sets the stage for a more profound comprehension of the intricate interplay between social structure and support provision within online communities.

## 2. Literature review

### 2.1. Social support provision: From support receiver to support provider

Scholars have dedicated significant efforts to social support research since the 1970s. Over the course of several decades, numerous studies have been conducted to gain insights into the factors that maximize the effects of social support [10,11], the various benefits it offers [12–14], the role of social networks in receiving social support [5], and the effects of one’s network position on receiving different types of social support [4,6]. Consistently, these studies have demonstrated that social support can significantly enhance the well-being of recipients [15], its effectiveness is particularly pronounced when the recipient is under high levels of stress [10], and the type of social support received is influenced by one’s position within the network [6]. While these studies have shed light on various aspects of social support, they have predominantly focused on the perspective of recipients who seek social support. Consequently, our understanding of the other side—the providers who supply social support—remains limited.

Social support exchange is analogous to a market transaction. Just as understanding the motivations and needs of both the demand and supply sides is crucial in comprehending market transactions, our understanding of social support exchange can be significantly enhanced by gaining a balanced understanding of both social support reception and provision. Specifically, our study aims to address the following inquiries: How does the social context or network, formed through social interactions and support exchanges, contribute to the promotion of social support provision? What types of social capital emerge within networked relationships, and how do they influence the provision of social support in OSGs?

Prior studies have underscored the significance of comprehending social support provision within the context of social factors, such as social norms [16], shared identity [17], homophily [18], and reciprocity [19]. Building on the premise that social components play an essential role in support provision, our research advances the field by examining social support provision through the lens of social capital theory, which provides a unified framework to understand how network structures generate resources that facilitate supportive behaviors.

### 2.2. Conceptualizing social support provision: Beyond quantity

This study focuses on informational and emotional supports. The literature on social support presents various types, with Cutrona and Suhr’s [20] taxonomy being the most universally adopted. This categorization delineates five functions of social support: informational, emotional, esteem, network, and tangible support. Within the realm of OSGs, informational and emotional supports emerge as the most common types of social support [21–24]. Informational support encapsulates the provision of advice, facts, and feedback, while emotional support entails expressing care, concern, empathy, and sympathy [20]. Although both types fundamentally aim to bolster individual well-being, they exhibit significant divergences in terms of motivation and mechanism [25,26]. Consequently, investigating the distinct theoretical trajectories that lead to these support behaviors is vital.

Social support provision is defined as the delivery of supportive actions or messages to others [27]. Prior research has predominantly concentrated on the quantitative aspect of social support provision [25], referencing the volume of social support an individual extends to others. Undoubtedly, the quantity of social support represents a crucial dimension in understanding this construct. However, a sole focus on quantity is insufficient to provide a comprehensive perspective on social support, including its origins and implications.

This study endeavors to expand the understanding of social support by incorporating two additional dimensions: quality and timing. The inclusion of quality and timing allows for a more nuanced examination of social support provision, aspects that have been previously overlooked in social support research that employs digital traces. Given that each dimension adds a unique facet to the concept of social support provision, scrutinizing each along with its antecedents elucidates the underlying mechanisms shaping these diverse dimensions of support provision.

The quality of informational support is conceptualized as the uniqueness of information contained within support messages provided by an individual. Within OSGs, members can offer supportive information either by replicating data from other sources or generating their unique or innovative insights. Information that merely duplicates other sources is seen as redundant and tends to hold less value for the recipients of the informational support compared to original insights [28,29]. Consequently, the uniqueness of information emerges as a crucial indicator of the quality of informational support within OSGs.

The quality of emotional support is characterized as the extent to which an emotional message is elaborated and reflects a support seeker’s expressions. According to the person-centeredness framework, the high quality of emotional support explicitly recognizes, elaborates, and investigates a support seeker’s feelings [30]. A message that mirrors a support seeker’s feelings aids them in gaining insight into their own emotions [30]. Furthermore, an expanded message that clarifies and validates a support seeker’s feelings enables them to assess their emotions in a broader context. Therefore, an elaborated message that reflect the support seeker’s expressions characterize the quality aspect of emotional support.

Another vital dimension considered in this study is timing, which pertains to the promptness of support provision. Although asynchronism of OSGs have positive aspects such as convenience for use [18] and the ability to accommodate responses from multiple users [17], empirical evidence reveals that users generally perceive immediate responses more positively than delayed ones [18,31]. Coping with stress is a dynamic process requiring different types of support at different times [32]. People seek social support “when” they need it, making a swift more value than a delayed one, as it better addresses the seeker’s immediate needs. Hence, timing is recognized as the third dimension of support provision.

In sum, this study focuses on the provision of informational and emotional support, and examining three underlying dimensions (quantity, quality, and timing) of each type. Given the distinct value of informational and emotional support, the uniqueness of information represents the quality of informational support, while the level of elaboration and reflection on support seekers characterizes the quality of emotional support. Table 1 presents definitions and examples of informational and emotional support drawn from the study data.

**Table 1:** Definitions and examples of informational and emotional support.

	Informational Support	Emotional Support
Definitions	A message that provides guidance or advice on solving a problem.	A message that provides empathy, comfort, or care to support and stabilize the recipient’s emotions.
Examples #1	I’ve heard that robotic surgery has fewer case studies and a higher risk compared to laparoscopic surgery, so I recommend laparoscopic surgery. As far as I know, the recovery process is almost the same for both.	Since it was detected early, your surgery should go well! Take care to avoid catching a cold. Wishing you a successful surgery—please update us afterward!
Examples #2	The reason saunas are discouraged for cancer patients is their weakened immune system, which increases the risk of infections. It’s best to avoid hot baths as well.	Never think that it won’t work out! Since you’re going back to your hometown, stay positive and believe it will happen—it really will!
Examples #3	Yes, that is a side effect of radiation therapy. These side effects gradually improve over time.	I feel like I can hear your mother’s tears. Wishing your father peace and rest in a place without pain. I also hope your mother finds comfort soon.

*Note*. The examples were sampled from the study data, with each example drawn from a different post.

### 2.3. Social capital theory: A structural perspective to understand social support provision

Studies investigating the precursors of social support provision in OSGs underscore the significance of social and relational perspectives. As discussed earlier, social factors play a crucial role in influencing social support provision [16–18,33,34]. This empirical evidence suggests that understanding the social context within OSGs offers valuable insights into the determinants that either encourage or limit social support provision.

Social capital theory postulates that individuals who are better connected with others tend to prosper [35]. The theory identifies two types of network structures that depict this “better connection,” namely brokerage [36] and closure [3]. Each of these structures generates different forms of social capital. Brokerage facilitates access to *non-redundant information* [35], while *trust* is cultivated within closed network structures [3]. As these network structures through their corresponding social capital promote individual’s competence and motivation that are necessary for social support provision [37], they have the potential to translate individual’s social connections into empowered supportive actions.

### 2.4. Characteristics of brokerage and its relationship with social support provision

Brokerage is conceptualized as a network structure where individuals bridge gaps between groups [35]. Those individuals, referred to as brokers, benefit from their positioning within structural holes that bridge gaps between weakly connected groups. As brokers maintain weak but diversified and non-redundant connections, they gain privileged access to a diverse pool of information that overlaps minimally. This position enables brokers to cultivate intellectual and emotional skills necessary for effective communication across a wide range of contacts [38]. Previous research has shown that brokers demonstrate elevated creativity [39], generate insightful ideas [40], and deliver unique information [28].

In the context of social support, it is predicted that brokerage facilitates the provision of both informational and emotional support. As previously noted, brokers have opportunities to develop intellectual and emotional competencies essential for effective support in both realms. Interacting with diverse individuals allows brokers to broaden their knowledge on a myriad of topics and acquire emotional skills useful for comforting individuals facing various challenges. Competency, or self-efficacy, is a key facilitator of behaviors [41]. With increased competence in informational knowledge and emotional skills, brokers are more likely to provide larger quantities of informational support [7] and emotional support. Furthermore, competence is a vital factor in delivering effective informational [25] and emotional support messages [37]. Therefore, brokers are expected to offer higher quality support in both areas than non-brokers. Moreover, by strategically positioning themselves within their social networks, brokers can efficiently exchange information and resources with others [35]. As a result, they may be better equipped to respond to the needs of their contacts more promptly than those not engaged in brokerage.

***H1a-c:*** *A brokerage position occupied by an individual user in an OSG will result in a) greater quantity, b) better quality, and c) quicker timing of informational support provision, compared to a position with less brokerage.*

***H2a-c:*** *A brokerage position occupied by an individual user in an OSG will result in a) greater quantity, b) better quality, and c) quicker timing of emotional support provision, compared to a position with less brokerage.*

### 2.5. Non-redundant information as social capital mediating brokerage and social support provision

The non-redundant information environment serves as a unique form of social capital that arises from a brokerage structure. As previously noted, a brokerage structure connects individuals from diverse backgrounds through weak ties, thereby facilitating exposure to a variety of non-overlapping information [35]. Although concrete empirical evidence remains sparse, prior research suggests that non-redundant information contributes to the generation of innovative ideas and the enhancement of creativity [42].

In the context of OSGs, the non-redundant information environment underpins the reason brokers can offer more effective informational support. Being embedded in such environments allows brokers to broaden their knowledge on diverse topics [35], which is a critical competency for providing informational support. Brokers are then equipped to offer unique informational support, as their placement in these environments encourages the generation of novel ideas by amalgamating various less overlapping pieces of information [39]. Since they are in a non-redundant information environment [43], brokers can discover or create this unique and valuable information more swiftly, leading to the prompt provision of informational support.

Similarly, a non-redundant information environment also facilitates brokers in delivering more effective emotional support. To offer effective emotional support, individuals need cognitive abilities to recognize others’ emotional and cognitive states and generate comforting messages [37]. Given that non-redundant information environments host a wider pool of information that one can use to develop such cognitive and social perception skills, compared to redundant information environments, they assist brokers in providing larger quantities and higher quality of emotional support promptly. Consequently, a positive relationship exists between brokerage and non-redundant information, and from non-redundant information to the diverse aspects of informational and emotional support provision, suggesting a mediating role of non-redundant information environments.

***H3a-c*** *: A non-redundant information environment will mediate the relationship between brokerage and a) the quantity, b) quality, and c) timing of information support provision.*

***H4a-c*** *A non-redundant information environment will mediate the relationship between brokerage and a) the quantity, b) quality, and c) timing of emotional support provision.*

### 2.6. Characteristics of closure and its relationship with social support provision

Closure represents a type of network structure characterized by strong connections among individuals [44], and strong ties often foster a sense of trust among network members [3]. This trustful environment encourages prosocial behaviors as individuals feel confident that their actions benefiting others will be reciprocated [3]. As members in closed networks are densely and directly connected, closure fosters frequent and direct communication among them, leading to shared values, mutual awareness of needs, and the fluid provision of social help [45].

Within the realm of social support, members embedded in closure are more likely to offer both informational and emotional support compared to those in less closed structures. Typically, closure comprises strong ties that spur a significant motivation for assistance [46]. Individuals connected through strong ties often exchange a wide array of social supports including companionship, minor services, and emotional support [26]. Empirical evidence indicates that people primarily receive social support on social media from close others with whom they share strong ties [47]. Bound by strong ties, members of a closed network are more likely to be motivated to provide both informational and emotional support, as they wish to alleviate any distress experienced by other members. Existing empirical studies validate the role of closure in fostering knowledge transfer and exchange, as well as emotional support [47–49].

Regarding the quality of social support, members in a closure are more likely to offer high-quality informational and emotional support. Despite the traditional notion that strong ties may not facilitate the provision of valuable information due to information redundancy, empirical evidence suggests that people often perceive information as more valuable when it comes from strong ties [50]. This is likely because individuals in a closed network develop mutual understanding and are capable of tailoring messages to fit the recipient’s prior knowledge and needs [51]. Following the same logic, members in closure should provide higher quality emotional support given their enhanced understanding of others’ emotional statuses and needs. Moreover, as members within closure care for others’ well-being and needs [52] and feel motivated to assist [46], closure will likely facilitate the prompt provision of social support when solicited.

***H5a-c*** *: A closure position occupied by an individual user in an OSG will result in a) greater quantity, b) better quality, and c) quicker timing of informational support provision, compared to a position with less closure.*

***H6a-c*** *: A closure position occupied by an individual user in an OSG will result in a) greater quantity, b) better quality, and c) quicker timing of emotional support provision, compared to a position with less closure.*

### 2.7. Trust as social capital mediating closure and social support provision

Trust is the form of social capital that can be derived from closed networks [3]. Within a closed network, members establish close relationships, leading to the development of trust. Trust has been demonstrated to play an important role in facilitating various social interactions, including monetary transactions in e-commerce [53], knowledge sharing in online communities [2], and support seeking [54].

**Fig 1:**
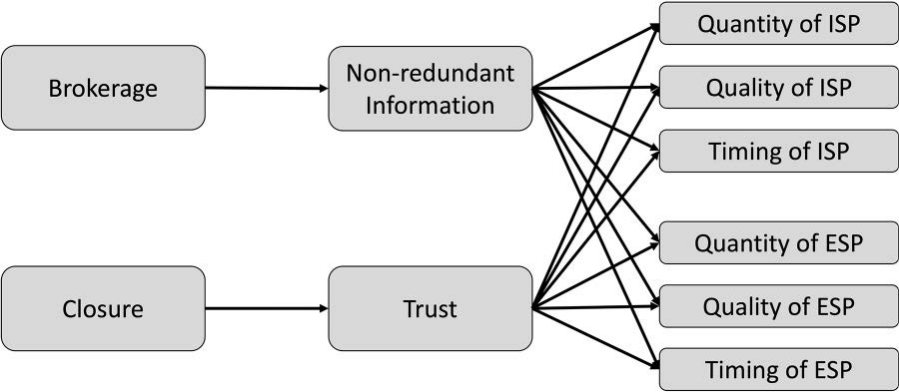
Proposed research model.

*Note*. ISP = Informational Support Provision; ESP = Emotional Support Provision

In the contexts of OSGs, trust could facilitate the provision of both informational and emotional social support. Members in a closed network are likely to be more motivated to provide such support, as they trust that their actions will be reciprocated when they themselves need support. Trust also impacts the quality and effectiveness of the support provided, as individuals are more inclined to offer genuine and beneficial assistance when they trust the recipient of their support. In a trusting environment, individuals feel more secure in sharing their emotional experiences [55]. The exchange of such affective self-disclosure can lead to a deeper understanding of each other’s emotional states, reinforcing the trusting relationships and thereby enhancing the quality of both informational and emotional support offered. Furthermore, trust can reinforce social obligations and the sense of responsibility to respond to support requests promptly [3], implying that members in a trustful network are more likely to offer social support in a timely manner. This discussion suggests a mediating role of trust in the relationship between closure and multiple dimensions of both informational and emotional support. Fig 1 illustrates the proposed research model.

***H7a-c*** *: Trust will mediate the relationship between closure and a) the quantity, b) quality, and c) timing of information support provision.*

***H8a-c*** *: Trust will mediate the relationship between closure and a) the quantity, b) quality, and c) timing of emotional support provision.*

The dynamic nature of OSGs with members constantly joining and leaving could play a significant role in how network structures and social capital influence social support provision. In the early stages of an OSG, members often have more opportunities to form close connections and establish trust. It is plausible that during this phase, closed network structures and trust may be more prominent [56], while brokerage structures and non-redundant information could be less prevalent. However, as the community grows and more members join, the dynamics between network structures, social capital, and social support provision may shift. For instance, brokerage positions might increase as new members join, and structural holes can be created amidst the network’s expansion. Consequently, the role of closed network and trust in support provision could be weaker in the later stage compared to the early stage. Understanding how these dynamics change as an OSG moves through different development stages—from early to growth and maturity stages—could provide crucial insights into the mechanism of support provision. Thus, this study proposes the following research question.

***RQ1*** *: Will the development stage of OSGs moderate the relationships among network structures, social capital, and social support provision?*

## 3. Method

### 3.1. Data collection and research design

Data were collected from a prominent online cancer community in South Korea. The Institutional Review Board (IRB) of Michigan State University in the U.S. approved the data collection with exemption (MSU Study ID: STUDY00003611). The IRB exempted the requirement for informed consent due to the archival nature of the data. Before the researchers accessed the data, each user’s screen name was replaced with a randomly generated six-digit number, and the original screen name was discarded to ensure full anonymity.

The programming language Python was employed to extract 12 years’ worth of data, spanning from August 13, 2007, to November 25, 2019. The data collection took three weeks, from November 26, 2019, to December 10, 2019. The gathered data included anonymized user screen names, user-generated posts and comments, the categories of these posts, the number of views each post received, and timestamps for all content. Data from the first two years (2007 and 2008) and the final year (2019) were excluded due to the community’s initial instability and the incompleteness of the final year’s data, respectively. The remaining data from 2009 to 2018 were employed for analysis.

The ideal research design to assess the proposed research hypotheses would be a panel design that could track the same group of users throughout the study period, from 2009 to 2018. However, due to the volatile nature of online communities, tracking the same set of users over a prolonged period presents significant challenges. Consequently, this study adopted an innovative design inspired by the revolving two-wave panel design utilized in survey research.

**Fig 2:**
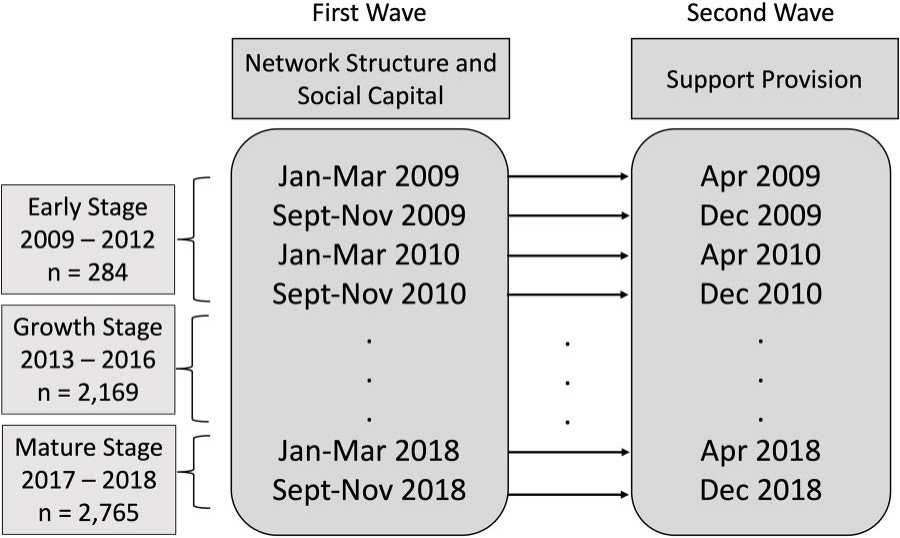
Study design.

As depicted in Fig 2, we generated 20 distinct panel datasets, with two panels per year: spring (January-April) and fall (September-December). Each panel dataset covers a four-month span. For each panel, we collected data in two sequential waves. In the first wave (January-March or September-November), we measured independent variables (brokerage and closure) and mediators (non-redundant information environment and trust). In the second wave (April or December), we measured dependent variables (quantity, quality, and timing of information support and emotional support). Data from May to August is omitted each year to minimize duplication within the panel datasets. While this interval does not entirely eliminate duplication, it serves to refresh the samples by creating a window for new members to join and existing members to dropout.

This research design carries three methodological advantages. First, the two-wave panel design enables the establishment of a temporal sequence between independent variables, mediators, and dependent variables, thereby enhancing the internal validity of the results. Second, the revolving panel design ensures a sufficient sample size for each panel, boosting the statistical power of the study. Lastly, the temporal order between different panels allows for explicit examination of how hypothesized relationships among variables may evolve over time.

The panel datasets were categorized into early stage (2009–2012), growth stage (2013–2016), and mature stage (2017–2018) with consideration of the community’s growth rate and to secure an adequate sample size for each stage so that we could investigate RQ1.

**Fig 3:**
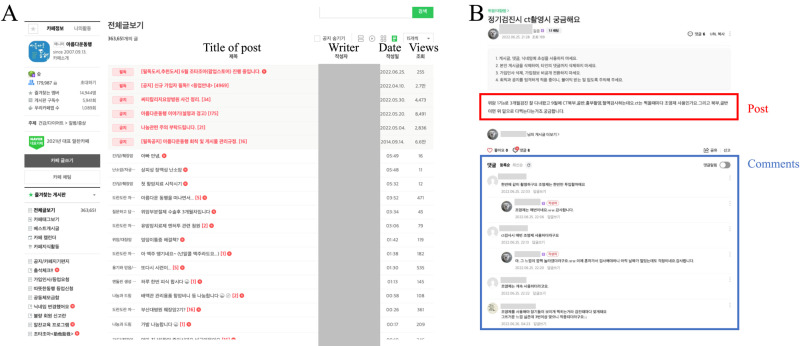
Screenshots of (A) community bulletin board and (B) post and comment page.

*Note*. Usernames are masked for privacy. Clicking a title of post in A directs to a page in B where users can see the post content and comments.

**Fig 4:**
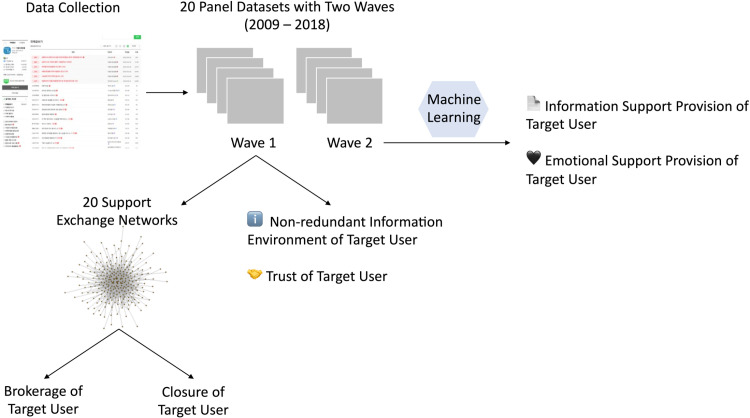
Workflow of data collection and processing.

The target users for the study were selected based on user activity and interaction with other users. S1 Appendix provides more details on sampling strategy. This selection method yielded valid sample sizes of 284 in the early stage, 2,169 in the growth stage, and 2,765 in the mature stage. Fig 3 provides screenshots of the online community to help readers’ understanding of the webpage structure. Fig 4 illustrates the workflow of data collection and processing.

### 3.2. Measurements

#### 3.2.1. Social support provision.

In this study, the provision of social support is conceptualized as a message drafted with the intent to assist others, whether it be informational, emotional, or in other ways. Informational support provision is characterized as a message that delivers advice or guidance to solve problems, while emotional support provision is defined as a message that offers comfort to another’s feelings through empathy, sympathy, or concern [11]. We employed KoBERT, a supervised machine learning tool [57], to distinguish between informational and emotional support provision. Additional details are available in S2 Appendix.

The provision of support can occur through either a post (i.e., initiating a discussion) or a comment in response to a post. Posts offering support are directed toward an unspecified large audience (e.g., providing information for general members), while comments offering support are tailored for a specific individual seeking social support. This study primarily focuses on the latter form, as it is the most prevalent type of support provision in the research context. According to the machine learning outcomes of this study, a significant 97% of support provision occurs through comments. Moreover, when a support provider offers multiple instances of informational support within a single thread, this study only analyzes the initial instance. This methodological decision was made because initial support instances typically address the original post author, while subsequent interactions may target various participants in the thread. Since our dataset lacks specific information about the intended recipients of these subsequent support instances, we restricted our analysis to only the initial instance of informational support from each provider. The same principle is applied to instances of emotional support.

##### Quantity and timing.

The quantity of informational support was measured by counting the number of information supports that a user provided during the specified study period (i.e., April for spring panel or December for fall panel). Similarly, the quantity of emotional social support was measured by counting the number of emotional supports. To alleviate skewness of data, the quantities of information and emotional support provision were log transformed.

The timing of informational support provision was measured by calculating the temporal gap between the request for informational support and its provision. For each target user, the average time difference across all threads in which the target user provided information support was utilized to denote the timing of informational support. To manage data skewness, the timing of informational support provision was also log-transformed. Subsequently, the log-transformed response time was reverse-coded by subtracting it from the maximum value. The purpose of this reverse coding was to align the interpretation of results with other dependent variables (i.e., the larger the value, the better). The timing of emotional support provision was measured in the same manner.

##### Quality of information support.

To measure the quality of information support, the uniqueness of an information support message was estimated [28]. A message is considered unique when its content is semantically independent from others. To quantify this, a Korean sentence BERT embedding model (KoBERT-embedding, hereafter) was employed to compute the semantic vector of an informational message [58]. Subsequently, a formula adapting cosine similarity was used to establish the semantic independence of an information support message offered by a focal user, in comparison with informational messages provided by others within the same thread. The overall quality of information support given by a focal person was assessed by averaging the uniqueness scores of each informational support they contributed. Further details can be found in S3 Appendix.

##### Quality of emotional support.

The quality of emotional support provision is operationalized in terms of a composite score based on the person-centeredness framework [30]. This score encapsulates both the degree of reflection of a support seeker’s sentiments and the degree of elaboration in the message. The KoBERT-embedding was used to compute the semantic vector of each message. The degree of reflection was evaluated by calculating the semantic similarity between the emotional support provided by a focal person and the initial support-seeking post, using cosine similarity for this computation. The degree of elaboration was assessed by counting the length of the emotional support message. The scores of reflection and elaboration were then multiplied to provide a measure of emotional support provision quality. The overall quality of emotional support given by a focal person was gauged by averaging these composite scores. More details are available in S4 Appendix, and S5 Appendix includes a validity assessment of text length as an indicator of elaboration.

#### 3.2.2. Network structure.

We construct a social support exchange network for each panel based on posting and commenting activities during the first wave among all users. Connections form when users exchange messages. For instance, if user A posts content and user B replies, A and B become connected. Similarly, if user C replies to A’s post, A and C are connected, but B and C remain unconnected since C’s intention was to interact with A specifically. Our study excludes reply-to-reply interactions due to absence of data indicating such interactions. To simplify the network analysis, we disregard the directionality of interactions while incorporating edge weights based on interaction frequency. The resulting social support exchange networks are weighted and undirected.

##### Brokerage.

This term refers to a structural position where an individual serves as a bridge between pairs of disconnected others [36]. Several metrics, such as betweenness centrality [59], network constraint [60], and effective size [36], have been proposed to quantify brokerage. In this study, betweenness centrality is chosen as the measurement for brokerage due to its ability to account for edge weight, thereby providing a richer understanding of the quality of bridging [61]. Betweenness centrality estimates the extent to which a focal person occupies the shortest paths between other actors [59]. While the shortest path in a binary network is determined by minimizing the number of intermediary nodes, the approach used in this study, Opsahl et al.’s method [61], measures the shortest path in a weighted network by considering both the number of intermediary nodes and edge weights. An R-package named bipartite was used to measure betweenness centrality in a weighted network [62]. To alleviate data skewness, betweenness centrality was log-transformed.

##### Closure.

This term represents a structural position in which an individual creates connections between others who are already connected [36]. Closure takes place when a tie is established to close a 2-length path to form a triangle, which is referred to as triadic closure [63]. A weighted local clustering coefficient was employed to measure closure. Unlike the unweighted local clustering coefficient, which simply calculates the proportion of triadic closure, the weighted version also accounts for edge weights [64], providing a more nuanced understanding of network relationships, such as frequency of social support exchanges. This study utilized Onnela et al.’s [65] method, which normalizes the edge weights based on the maximum weight in the network. An R-package called DirectedClustering was used to measure each node’s closure structure using Onnela’s method. To reduce data skewness, closure was log-transformed.

#### 3.2.3. Social capital.

##### Non-redundant information environment.

A focal individual’s non-redundant information environment is characterized by their exposure to diverse and distinct information while they interact with other members. This study makes certain assumptions to pare down others’ messages to which a focal person may be exposed. When a focal person posts an update, we presuppose that they would be exposed to all comments, as all comments are directed at the focal person. When a focal person comments on a post authored by another person, it is assumed that the focal person would be exposed to the post and any comments made prior to their own. The KoBERT-embedding was employed to compute the semantic vector of messages. Then, adapting the operationalization of non-redundancy information proposed by Aral & Dhillon [28], we compiled the messages that a focal person is presumably exposed to and computed semantic difference between each pair of the messages, using an adapted cosine similarity. The average semantic differences across messages were used to indicate the non-redundant information of a focal person. A higher value indicates that, on average, the focal individual is exposed to less overlapping messages. Further details are available in S6 Appendix.

##### Trust.

This was measured using a composite index of self-disclosure and reciprocity. These are behaviors individuals typically exhibit when they have trust in others [25,66]. Self-disclosure was quantified by calculating the proportion of self-disclosure-related words at the content level [25]. These proportions were then averaged at the user level to represent a focal individual’s level of self-disclosure. Reciprocity was quantified by calculating the proportion of reciprocal interactions a focal user engaged in. Unlike brokerage and closure measures, we accounted for the directionality of interactions to accurately capture mutual exchanges. Both self-disclosure and reciprocity were normalized and then incremented by a small constant (0.01) to prevent the resultant product from becoming 0 if either value is 0. The product of these scores represents the level of trust for a focal individual. Further details are available in S7 Appendix.

#### 3.2.4. Control variable.

Individual post activity (i.e., initiating discussion) was included as a control variable. This activity is quantified by tallying the number of posts a focal individual uploaded during the four months of the study period. To reduce skewness, this control variable was log-transformed. Table 2 provides an overview of the study variables and their operationalizations.

**Table 2:** Study variables and operationalizations.

Variables	Operationalizations	Data Types
**Dependent Variables**		
Quantity of ISP	The total number of ISP that a focal person provided	–
Quality of ISP	Averaged semantic independence between a focal person’s ISP content and other ISP content across threads that a focal person participated in	Semantic
Timing of ISP	Averaged time difference between a focal person’s ISP content and support-seeking content across threads that a focal person participated in	Temporal
Quantity of ESP	The total number of ESP that a focal person provided	–
Quality of ESP	A composite score of semantic similarity between focal person’s ESP content and a support seeker’s content and the length of the focal person’s ESP content. Then, the composite index averaged to represent quality of ESP	Semantic
Timing of ESP	Averaged time difference between a focal person’s ESP content and support-seeking content across threads that a focal person participated in	Temporal
**Independent Variables**		
Brokerage	Weighted betweenness centrality	Relational
Closure	Weighted local clustering coefficient	Relational
**Mediators**		
Non-redundant Information Environment	Averaged semantic independence across messages that a focal person was exposed to	Semantic
Trust	A composite index of self-disclosure and reciprocity that a focal person displayed	Semantic and Relational
**Controls**		
Post activities	The number of posts that a focal person uploaded during the study period	–

*Note.* ISP = Information Support Provision; ESP = Emotional Support Provision

### 3.3. Analytic plans

To test the research hypotheses and the research question, we conducted two multigroup path analyses using the R-package lavaan [67]. Initially, we constructed a coefficient-constrained multigroup model to evaluate the research hypotheses. This model assumes that loadings (or coefficients) remain identical across groups (i.e., the early, growth, and mature stages of the community). This approach enables the researcher to estimate the general associations among variables, disregarding the group factor.

To investigate the proposed research question, we constructed a coefficient-free multigroup model and compared it to the coefficient-constrained multigroup model. Unlike the coefficient-constrained model that sets fixed loadings across groups, a coefficient-free multigroup model allows groups to exhibit different coefficients. By comparing the coefficient-free model with the coefficient-constrained model via the chi-square difference test, we can determine whether path effects statistically vary across groups. In essence, the comparison between these two models provides the opportunity to examine whether the paths are moderated by the group factor.

## 4. Results

### 4.1. Descriptive results

The total sample size of the data is 5,218. The sample consists of 3,574 unique users, 73% (*n* = 2,607) of which appear once and 27% (*n* = 967) of which appear more than once across panel datasets. On average, the target individuals updated 5 posts (*SD* = 8.50, median = 3) and 47 comments (*SD* = 108.39, median = 17) during the four months of the study period. Among uploaded content, 51% of them are support provision, 12% are support seeking, 37% are others (e.g., gratitude expression). Among support-provision content, 48% are information support and 36% are emotional support, which indicates that information and emotional support provision (85%) are the dominant types of social support compared to other types of social support (15%).

Descriptive network statistics of the OSG at different development stages help understand the study context. It is notable that the data for the following descriptive statistics are based on activities from not only the target users but also other members who were active and potentially interacted with target members during the study period. The average network size in the early stage was about 477, and it increased to 2,337 in the growth stage and to 6,947 in the mature stage. Although on average a member’s personal network expanded along with the growth of the community, the expansion did not increase after reaching a certain level. The degree centrality metrics indicate that a member interacted with 7 other members on average in the early stage, and the number expanded to 13 in the growth stage. However, despite the continuing growth, in the mature stage, the degree centrality remained at 12, which is similar to that of the growth stage.

Table 3 provides more detailed statistics of the network metrics along with other network metrics such as brokerage, density, and closure that can provide more information about the context of the OSG across the study panels. Table 4 provides descriptive statistics such as mean, standard deviation, and zero-order correlation of the study variables.

**Table 3:** Summary of network statistics across the panel.

Study Time	Community Level		Individual Level		
Network Size	Density	Brokerage^a^	Closure^a^	Degree Centrality
Early (1–8)	477.25	0.011	667.052	0.013	6.812
Growth (9–16)	2337.50	0.005	4540.174	0.003	13.457
Mature (17–20)	6946.75	0.001	15851.119	0.001	12.444
1 (2009 Spring)	205	.031	194.105	.018	10.020
2 (2009 Fall)	380	.014	484.468	.021	8.695
3 (2010 Spring)	404	.011	519.681	.018	7.243
4 (2010 Fall)	482	.007	734.693	.018	5.203
5 (2011 Spring)	417	.008	621.144	.007	5.247
6 (2011 Fall)	491	.006	686.347	.007	4.925
7 (2012 Spring)	798	.004	1145.271	.007	5.241
8 (2012 Fall)	641	.009	950.710	.007	7.922
9 (2013 Spring)	841	.009	1369.404	.004	10.792
10 (2013 Fall)	1458	.008	2407.821	.005	15.733
11 (2014 Spring)	1697	.006	3043.873	.003	15.414
12 (2014 Fall)	2369	.004	4414.349	.003	15.0120
13 (2015 Spring)	2555	.003	4701.388	.003	12.200
14 (2015 Fall)	2856	.003	5413.802	.004	12.812
15 (2016 Spring)	3353	.003	6861.271	.001	13.368
16 (2016 Fall)	3571	.002	8109.480	.001	12.318
17 (2017 Spring)	5315	.002	11378.600	.002	13.104
18 (2017 Fall)	6374	.001	14282.172	.001	12.980
19 (2018 Spring)	7514	.001	17993.939	.001	12.206
20 (2018 Fall)	8584	.001	19749.766	.001	11.486

*Note.* Statistics of the early stage are obtained by averaging statistics of panels 1–8 in study time; statistics of the growth stage are obtained by averaging statistics of panel study time 9–16 in study time; and statistics of the mature stage are obtained by averaging statistics of panel 17–20 in study time. The first batch of each panel data (January–March or September–November) is used for the descriptive statistics.

^a^Raw scores of brokerage and closure are reported. Log-transformed scores of brokerage and closure are used in the main analysis.

**Table 4:** Means, standard deviations, and zero-order correlations of variables.

			Dependent Variables						Independent Variables		Mediators		Control
	*M*	*SD*	1	2	3	4	5	6	7	8	9	10	11
1^a^	1.44	0.76	1.00										
2	0.53	0.08	.003	1.00									
3 ^a^	8.71	1.49	.14^***^	.05^***^	1.00								
4 ^a^	1.39	0.73	.50^***^	.01	.10^***^	1.00							
5	0.22	0.11	.06^**^	−.12^***^	−.06^***^	−.13^***^	1.00						
6 ^a^	9.48	1.49	.12^***^	.03	.35^***^	.09^***^	−.06^***^	1.00					
7 ^a^	6.58	4.42	.40^***^	.04^**^	.10^***^	.30^***^	.002	.08^***^	1.00				
8 ^a^	0.003	0.01	−.06^***^	−.06^***^	−.12^***^	.02	.04^**^	−.14^***^	−.08^***^	1.00			
9	0.64	0.05	.33^***^	.11^***^	.11^***^	.11^***^	.07^***^	.14^***^	.33^***^	−.06^***^	1.00		
10	0.07	0.04	−.02	−.04^**^	.01	.02	.10^***^	.05^**^	.25^***^	.05^**^	.15^***^	1.00	
11 ^a^	1.30	1.03	.34^***^	−.01	.06^***^	.28^***^	.01	.07^***^	.57^***^	−.01	.28^***^	.35^***^	1.00

*Note*.

* *p* < .05;

** *p* < .01;

*** *p* < .001

^a^These variables are log-transformed.

Dependent Variables: 1 = Information Support Quantity; 2 = Information Support Quality; 3 = Information Support Timing; 4 = Emotional Support Quantity; 5 = Emotional Support Quality; 6 = Emotional Support Timing.

Independent Variables: 7 = Brokerage; 8 = Closure.

Mediators: 9 = Non-redundant Information Environment; 10 = Trust.

Control: 11 = Post Activities.

### 4.2. Hypotheses testing

The proposed research model fitted to the data (χ^2^(77) = 359.63, *p* < .001, CFI = .97, RMSEA = .05, SRMR = .03). The results largely supported the hypotheses suggesting that brokerage facilitates informational support provision via non-redundant information environment, whereas there were mixed findings about the role of brokerage in promoting emotional support provision. In addition, although closure played a restricted role in promoting both informational and emotional support provision, closure facilitated a high quality of emotional support via trust.

H1a, H1b, and H1c examined the direct effect of brokerage on information support provision. Consistent with H1a, individuals provided more information support (*β* = .25, *p* < .001) when they were embedded in a brokerage position. Inconsistent with H1b and H1c, brokerage did not directly facilitate the provision of unique information (*β* = .03, *p* = .08) and the timing of information support (*β* = .03, *p* = .06). Therefore, H1a was supported, whereas H1b and H1c were rejected.

H2a, H2b, and H2c predicted the positive direct effect of brokerage on emotional support provision. Consistent with H2a, individuals provided more emotional support when they were in a higher level of brokerage position (*β* = .22, *p* < .001). However, inconsistent with H2b and H2c, brokerage did not have direct effect on the quality (*β* = −.03, *p* = .13) and the timing of emotional support provision (*β* = −.01, *p* = .52). Therefore, H2a was supported, whereas H2b and H2c were rejected.

H3a, H3b, and H3c examined the mediating role of non-redundant information in informational support provision. Consistent with the hypotheses, a non-redundant information environment mediated the relationships between brokerage and the quantity (*β* = .06, *p* < .001), brokerage and the uniqueness (*β* = .03, *p* < .001), and brokerage and information support timing (*β* = .02, *p* < .001). Therefore, H3a, H3b, and H3c were supported.

H4a, H4b, and H4c concerned the mediating role of non-redundant information environment in emotional support provision. Consistent with H4b and H4c, a non-redundant information environment mediated the impact of brokerage on the quality of emotional support (*β* = .02, *p* < .001) and the timing of emotional support (*β* = .03, *p* < .001). Inconsistent with H4a, the impact of brokerage on the timing of emotional support was not mediated by a non-redundant information environment (*β* = −.003, *p* = .39). Therefore, H4b and H4c were supported and H4a was rejected.

H5a, H5b, and H5c predicted the positive direct effect of closure on informational support provision. Despite the significant result, closure did not have a positive direct impact on the quantity (*β* = −.02, *p* < .05), which was inconsistent with H5a. Moreover, inconsistent with H5b and H5c, closure did not have direct impacts on the uniqueness (*β* = .002, *p* = .84) and the speed of information support (*β* = .01, *p* = .41). Thus, H5a, H5b, and H5c were rejected.

H6a, H6b, and H6c predicted that the positive direct effect of closure on emotional support provision. Consistent with H6a, closure had a direct effect on the quantity of emotional support (*β* = .03, *p* < .001). Inconsistent with H6b, closure had a negative impact on the quality of emotional support (*β* = −.03, *p* < .05). Moreover, inconsistent with H6c, closure did not affect the speed of emotional support (*β* = −.01, *p* = .21). Therefore, H6a was supported and H6b and H6c were rejected.

H7a, H7b, and H7c concerned the mediating role of trust in information support provision. Inconsistent with H7a and H7b, there were negative indirect effects on the quantity (*β* = −.01, *p* < .001) and the uniqueness of information support provision (*β* = −.004, *p* < .01). Furthermore, inconsistent with H7c, trust did not mediate the relationship between closure and the speed of information support provision (*β* = −.001, *p* = .18). Thus, H7a, H7b, and H7c were rejected.

H8a, H8b, and H8c predicted the mediating role of closure in emotional support provision. Consistent with H8b, closure mediated the impact of trust on the quality of emotional support (*β* = .01, *p* < .001). Inconsistent with H8a, the impact of closure on the quantity of emotional support was negatively mediated by trust (*β* = −.01, *p* < .001). Moreover, inconsistent with H8c, trust did not mediate the relationship between closure and the speed of emotional support (*β* = .001, *p* = .22). Thus, H8b was supported and H8a and H8c were rejected. Table 5 provides the summary of the results.

**Table 5:** Results of hypotheses testing.

Hypotheses	Relationships	Beta	Supported?
H1a	Brokerage → Info quantity	.245^***^	Yes
H1b	Brokerage → Info quality	.029	No
H1c	Brokerage → Info timing	.034	No
H2a	Brokerage → Emo quantity	.222^***^	Yes
H2b	Brokerage → Emo quality	−.027	No
H2c	Brokerage → Emo timing	−.011	No
H3a	Brokerage → Non-redundancy → Info quantity	.057^***^	Yes
H3b	Brokerage → Non-redundancy → Info quality	.027^***^	Yes
H3c	Brokerage → Non-redundancy → Info timing	.016^***^	Yes
H4a	Brokerage → Non-redundancy → Emo quantity	−.003	No
H4b	Brokerage → Non-redundancy → Emo quality	.022^***^	Yes
H4c	Brokerage → Non-redundancy → Emo timing	.028^***^	Yes
H5a	Closure → Info quantity	−.020^*^	No
H5b	Closure → Info quality	.002	No
H5c	Closure → Info timing	.009	No
H6a	Closure → Emo quantity	.034^***^	Yes
H6b	Closure → Emo quality	−.025^*^	No
H6c	Closure → Emo timing	−.014	No
H7a	Closure → Trust → Info quantity	−.013^***^	No
H7b	Closure → Trust → Info quality	−.004^**^	No
H7c	Closure → Trust → Info timing	−.001	No
H8a	Closure → Trust → Emo quantity	−.008^***^	No
H8b	Closure → Trust → Emo quality	.008^***^	Yes
H8c	Closure → Trust → Emo timing	.001	No
**R-squared Values of Variables**			
Info quantity		.237	
Info quality		.010	
Info timing		.007	
Emo quantity		.138	
Emo quality		.020	
Emo timing		.016	
Non-redundancy		.093	
Trust		.150	
**Model Fit**			
Chi-square (*df* = 77)		350.63^***^	
CFI		.97	
RMSEA		.05	
SRMR		.03	

*Note.*

* *p* < .05;

** *p* < .01;

*** *p* < .001

Info quantity = Information support quantity; Info quality = Information support quality; Info timing = Information support timing; Emo quantity = Emotional support quantity; Emo quality = Emotional support quality; Emo timing = Emotional support timing; Non-redundancy = Non-redundant information environment

### 4.3. Research question testing

RQ1 asks if paths from network structure, social capital, to social support provision differ across the development stages of the OSG. To examine the research question, a coefficient-free multigroup model was compared with a coefficient-constrained multigroup model via the chi-square difference test. The two models had a significant difference such that the fit of a coefficient-free model is better than that of a coefficient-constrained model, indicating that path coefficients are moderated by the development stage of the OSG, χ^2^ difference (62) = 269.38, *p* < .001. Thus, the relationships between network structure, social capital, and social support provision varied across the early, growth, and mature stages of the community.

Overall, the findings highlight that the roles of brokerage and closure and their social capital in facilitating social support provision are more critical in the later stages than the early stage. For instance, the indirect effect of brokerage via non-redundant information environment on information quantity, uniqueness, and speed were not significant in the early stage whereas these indirect effects were significant in the growth and mature stages. Similarly, the indirect impacts of brokerage via non-redundant information environment on the quality and speed of emotional support provision were not significant in the early stage but were significant in the growth and mature stages.

The roles of closure and trust in promoting information and emotional support were nuanced and complex. As the results of hypotheses suggested, many results were nonsignificant or contradictory to the predictions. Nevertheless, some significant results indicated that closure and trust play important roles in emotional support provision especially in the later stages of OSG. The direct impact of closure on the quantity of emotional support was not significant in the early stage but significant in the later stage. Moreover, the indirect impact of closure via trust on the quality of emotional support was only significant in the growth and mature stages. Table 6 summarizes path coefficients at different stages of the OSG.

**Table 6:** Path coefficients at the early, growth, and mature stage of the OSG.

Relationships	Early Stage (*n* = 284)	Growth Stage (*n* = 2,169)	Mature Stage (*n* = 2,765)
Brokerage → Info quantity	.239^***^	.247^***^	.247^***^
Brokerage → Info quality	.068	.004	.048^*^
Brokerage → Info timing	.066	.013	.054^*^
Brokerage → Emo quantity	.146^*^	.264^***^	.239^***^
Brokerage → Emo quality	−.109	.005	−.054^*^
Brokerage → Emo timing	.144^*^	−.044	.003
Brokerage → Non-redundancy → Info quantity	.017	.056^***^	.065^***^
Brokerage → Non-redundancy → Info quality	.004	.035^***^	.026^***^
Brokerage → Non-redundancy → Info timing	.021	.013^*^	.016^**^
Brokerage → Non-redundancy → Emo quantity	.011	.006	−018^**^
Brokerage → Non-redundancy → Emo quality	−.018	.013^*^	.038^***^
Brokerage → Non-redundancy → Emo timing	.017	.032^***^	.024^***^
Closure → Info quantity	−.014	−.057^**^	−.031
Closure → Info quality	−.003^*^	−.000	−.000^*^
Closure → Info timing	.003^*^	.000	.000
Closure → Emo quantity	.043	.109^***^	.053^**^
Closure → Emo quality	−.014	−.067^**^	−.028
Closure → Emo timing	−.044	−.026	−.008
Closure → Trust → Info quantity	.003	−.026^***^	−.023^***^
Closure → Trust → Info quality	−.002	−.006	−.009^**^
Closure → Trust → Info timing	.001	−.004	−.002
Closure → Trust → Emo quantity	.002	−.015^***^	−.018^***^
Closure → Trust → Emo quality	.001	.014^***^	.020^***^
Closure → Trust → Emo timing	.001	.004	.002
**R-squared Values of Variables**			
Info quantity	.271	.258	.254
Info quality	.021	.018	.017
Info timing	.097	.006	.009
Emo quantity	.199	.160	.110
Emo quality	.048	.014	.039
Emo timing	.067	.019	.012
Non-redundancy	.066	.117	.135
Trust	.116	.142	.141
**Model Fit**			
Chi-square (*df* = 15)	81.25^***^		
CFI	.99		
RMSEA	.05		
SRMR	.01		

*Note.*

* *p* < .05;

** *p* < .01;

*** *p* < .001.

Info quantity = Information support quantity; Info quality = Information support quality; Info timing = Information support timing; Emo quantity = Emotional support quantity; Emo quality = Emotional support quality; Emo timing = Emotional support timing; Non-redundancy = Non-redundant information environment

## 5. Discussion

This study examines social support provision in an online cancer community through the lens of social capital theory. The results reveal that brokerage and non-redundant information environment effectively foster various dimensions of informational and emotional support provisions. Conversely, closure and trust show mixed outcomes in their facilitation of informational and emotional support provisions. While closure and trust aid in delivering high-quality emotional support, they either have negative impacts or no significant effects on other aspects of social support. The findings also highlight the significance of acknowledging the dynamic nature of OSGs by illustrating that the influence of network structures and social capital on support provision changes in relation to the OSG’s development. Overall, these findings suggest that social capital theory offers a valuable framework for elucidating the social pathways that lead to informational and emotional support provisions.

### 5.1. Study findings and implications

The findings align with previous studies asserting that individuals in high brokerage positions expand their knowledge and make informational contributions by capitalizing on non-redundant information [42] (H1a–c and H3a–c). These findings support the concept that bridging social connections or interactions empower individuals to access non-duplicative information and promote the circulation of knowledge [35]. While the social support literature has proposed the advantages of OSGs for information support based on the concept of weak ties [17], empirical evidence backing this argument has been sparse, leaving the specific mechanism vague. The findings from this current study clarify why OSGs can be a crucial source for information support. OSGs serve as a useful source of information support because individuals leverage the weak-tie nature of OSGs. By being exposed to a non-redundant information environment, those in brokerage positions are capable of offering more ample, prompt, and valuable information support to community members.

The findings of this study show that brokerage and the associated social capital not only enhance informational support provision but also improve emotional support provision (H2a-c and H4a-c). Previous research has primarily explored the role of brokerage and non-redundant information in the context of informational contributions [28,39,40]. This study is among the first to investigate the role of brokerage and non-redundant information in the context of emotional support. The results of the study back up the claim that an individual’s cognitive ability and social perception are crucial in providing effective emotional support [37], and such cognitive competency can be learned and developed by being embedded in diverse and less redundant information environments.

In contrast to the study hypotheses, the closure structure and trust hinder information support provision (H5a-c and H7a-c). These findings are inconsistent with theories suggesting that closure structures mobilize social assistance with enhanced motivation [45,46]. It seems that the helping motivation derived from social relationships is not an adequate qualification for information support provision. Indeed, relational intimacy does not guarantee informational support provision [68]. Prior empirical studies indicate that relational attributes (e.g., frequent interaction, trust, social identity) do not have a significant impact on informational support provision [25]. It seems that rather than relational closeness or strength, knowledge or cognitive abilities, which increase an individual’s capacity to provide information, could play a more crucial role in informational support provision [25,37]. While individuals in a closure structure may possess strong motivation to help others, they might lack the required abilities or resources to do so, possibly due to their embeddedness in redundant information environments.

Closure facilitates the provision of high-quality emotional support through trust (H8b) rather than having a direct effect on its own (H6b). These results align with prior research suggesting that individuals within trustful relationships effectively fulfill one another’s emotional support needs through compassionate and responsive communication [69]. It appears that individuals who trust their fellow members in OSGs are more likely to provide emotionally responsive and high-quality messages. The quality of supportive messages has been correlated with positive outcomes for the recipients [30], thereby making the identification of factors that enhance the provision of high-quality messages a crucial issue in the field of social support research. While previous studies have identified relational factors as significant influencers of the quality of emotional support [70], this study expands on this body of research by explicitly specifying trust as a key underlying mechanism that allows individuals in close relationships to provide high-quality emotional support. Therefore, fostering trust within online communities may be an effective strategy for improving the quality of emotional support shared among its members.

Although closure and trust play a role in facilitating a higher quality of emotional support, the effect was relatively small. Moreover, many of the proposed research hypotheses concerning the relationships between closure, trust, and emotional support were rejected (H6b, H6c, H8a, and H8c), implying limited effects of closure and trust on emotional support provision. This limited role could be attributed to the nature of OSGs, which largely revolve around weak-tie relationships. As the descriptive statistics of this study indicate, closure is not a prevalent social structure at least within the studied OSG. Given the scale of such communities, where countless members are posting and interacting daily, sustaining long-term interactions with the same group of individuals may be a difficult endeavor [17,18]. Furthermore, the existence of a closed network can be easily threatened by the withdrawal of even a single member. Consequently, maintaining a closure structure over an extended period in OSGs can be highly challenging. The nature of computer-mediated communication environments, which are typically characterized by a lack of social cues and potential delays in responses, may further hinder the establishment of close-knit networks and the development of intimate relationships. Therefore, it is possible that the weak-tie nature of OSGs could render closure structures fragile and less impactful, which in turn could dilute the effects of closure and trust on the various aspects of emotional support.

The findings underscore the importance of considering the unique characteristics and developmental stages of OSGs when assessing the dynamics of network structure, social capital, and support provision (RQ1). Specifically, this study found that as OSGs develop and grow, their networks tend to diversify, characterized by an increase in brokerage and a decrease in closure. This implies that these groups tend to embody the “weak-tie” nature of social relationships more strongly over time. As the size of the network expands, members have increased opportunities to interact with new individuals rather than continuously engaging with the same people. This dynamic enhances the weak-tie nature of OSGs and appears to confer benefits to the community as a whole. In comparison to the early stages of an OSG, the impacts of brokerage and non-redundancy on both informational and emotional support provision are found to be greater in the growth and mature stages. This suggests that the enhanced weak-tie nature of the group amplifies the impact of weak-tie-related structures and social capital on social support.

Interestingly, as the weak-tie network within the OSG becomes more pronounced, the importance of closure and trust—elements rooted in strong-tie relationships—increases, particularly for emotional support provision. During the early stage of the OSG, where members share relatively close relationships, the role of closure and trust in emotional support may have been less pronounced. However, as the OSG expands and welcomes an influx of new members, maintaining strong-tie-based structures becomes increasingly important for amplifying the quantity of emotional support, while trust cultivation enhances the quality of support provided. This underscores the value of nurturing and sustaining strong-tie relationships and trust within the OSG [51], particularly as OSGs continue to grow and reinforce the weak-tie nature of their networks.

### 5.2. Theoretical and practical contributions

This study offers significant theoretical and practical contributions. It enriches the social capital literature by emphasizing the benefits of distinctly separating social capital from network structures. Despite their conceptual differences, many previous studies have treated social capital as synonymous with network structures [25, 71]. However, recent evidence suggests that network structures such as brokerage should not be used as a proxy for social capital (e.g., access to non-redundant information), given their conceptual and empirical differences [72]. This conceptual reductionism, which conflates network structure with social capital, obstructs our understanding of the theoretical pathway from network structure, through social capital, to social behaviors. The findings of the current study validate that network structures and social capital are distinct entities. By distinguishing these two concepts, we can enhance research by elucidating the theoretical trajectories leading to prosocial behaviors.

This study enhances the social support literature by conceptualizing the provision of social support as a multifaceted concept. As opposed to the development of other perceptual support concepts, the conceptual and operational definitions of behavioral support have seen minimal development. Consequently, most previous studies focus solely on the quantity aspect of social support provision [25]. This limitation can result in neglecting crucial mechanisms of support provision. For instance, had the current study only measured the quantity of support provision, it would have failed to uncover the role of closure and trust in promoting the quality of emotional support. By consolidating the aspects of quantity, quality, and timing—which collectively offer vital behavioral information about support provision—this study yields meaningful insights into social support provision.

Practically, this study encourages community administrators and web designers to devise strategies for enhancing social connections among members, drawing upon theoretical guidance and empirical evidence from social capital research. Given that user interactions are significantly influenced and directed by the functions and services provided by OSGs, administrators and web designers have the power to foster network configurations and social capital. For the longevity of OSGs, it’s essential to strike a balance between weak and strong ties to facilitate both informational and emotional support provisions. However, considering the inherent weak-tie nature of OSGs, these groups may need to introduce features and services that can bolster strong-ties-based network structures (e.g., closure) and social capital (e.g., trust) among members. For instance, they could establish a sub-community system, enabling a small group of members to engage in intensive discussions on specific topics.

### 5.3. Limitations and future study directions

This study has limitations that pave the way for future research directions. A primary limitation of this study is the focus on a single communication channel (i.e., online support community). Given that people use multiple communication channels for social support exchange [73], this narrow observation may exclude support behaviors occurring outside the community platform. Moreover, as people tend to use different communication channels as their relationship with others develops [74], they may engage in social support exchange via other personal communication channels as their relationships with fellow community members progress. Therefore, future research should incorporate multiple communication channels individuals use for social support exchange to gain a more comprehensive understanding of the role of networks and social capital in support provision.

This research is limited in providing nuanced insights into support provision that differentiate between closely connected group members and more distant connections. Individuals in closure networks may distinguish between members within their immediate social circle and those outside it when providing social support, and patterns of support may differ depending on the recipient. For example, individuals in closure networks may provide greater amounts and higher quality of social support more promptly to members of their inner circles compared to those at the periphery of their networks. This nuanced distinction may explain why this study failed to find significant results for most pathways linking closure and social support. Future research should explore this nuanced approach to fully understand the social mechanisms underlying social support provision.

Finally, this research constructed a social network based on interactions rather than relationships, which limits the generalizability of our findings to relationship-based networks (e.g., closeness or friendship). Although interaction frequency and relational intimacy are related, they are not equivalent. For instance, individuals who interact frequently do not necessarily maintain close relationships, nor does infrequent interaction always indicate relational distance. This implies that brokerage and closure measured in this research could be qualitatively different from those measured through a relationship-based network. Future research should replicate our study using relationship-based social networks where the closeness between individuals is explicitly known.

## 6. Conclusion

The present study conducted an in-depth investigation of how network structures and social capital emerging from these networks foster the provision of informational and emotional support within an OSG. Leveraging computational methodologies, the study collated data from an online cancer community, measured the network, social capital, and various facets of social support provision, and analyzed the proposed research model. The findings underline that a brokerage structure and non-redundant information enhance the quantity, quality, and timely provision of both informational and emotional support. Although the closure structure and trust do foster higher-quality emotional support provision, the overall findings suggest their impacts remain relatively limited in promoting social support within the context of OSGs. Additionally, the social mechanisms linking networks to social capital and social capital to support provision vary based on the OSG’s stage of development. By elaborating on the social mechanisms that lead to informational and emotional support provision, and considering their dynamic nature, the study significantly deepens our understanding of the theoretical associations among network structure, social capital, and social support provision.

## Supporting information

S1 Appendix Data cleaning and sampling. (DOCX)

S2 Appendix Social support provision: Manual coding and machine leaning. (DOCX)

S3 Appendix Measurement of information support quality. (DOCX)

S4 Appendix Measurement of emotional support quality. (DOCX)

S5 Appendix Validity assessment of text length as an indicator of emotional support elaboration. (DOCX)

S6 Appendix Measurement of non-redundant information environment. (DOCX)

S7 Appendix Measurement of trust. (DOCX)
