# Supplementary material for: Bridging and bonding: The roles of brokerage and closure in mobilizing support provision in online support groups
Source: PLoS One. 2025 Jun 10;20(6):e0325108. doi: 10.1371/journal.pone.0325108 (PMC12151367; doi:10.1371/journal.pone.0325108)
Supplement: S1 Appendix — (DOCX) [file pone.0325108.s001.docx]

**Bridging and Bonding: The Roles of Brokerage and Closure in Mobilizing Support Provision in Online Support Groups**

**Supplemental Materials**

**S1 Appendix. Data Cleaning and Sampling**

The collected data consisted of 208,261 posts and 1,998,135 replies in total. Any unwarranted artifacts, such as empty content, misaligned data, and duplications, that occurred due to technical glitches during the data collection process were addressed. Specifically, posts and comments encompassing empty or misaligned data were discarded, and in the case of duplications, only distinct data were preserved. After data cleaning, the count of posts and replies was trimmed down to 204,720 and 1,954,209, respectively. A cumulative total of 54,207 unique users had posted at least one comment or one post in the OSG.

A valid sample was curated based on user activity and interactions with other users. In order to measure each user's network structures, social capital, and support provisions, a certain level of activity and interaction was necessary. Hence, two criteria were applied to select target users. Firstly, a user needed to interact with at least two other members in the first wave of panel data, which was utilized to measure network structure and social capital. Secondly, the same user was required to provide at least one instance of informational and emotional social support in the second wave of panel data, which was employed to measure support provision. In instances where a user appeared across multiple panel datasets - for instance, users who featured in both the January-April 2010 and September-December 2010 datasets - there were two possible options: treating each appearance as an independent sample or discarding them altogether. We opted for the former approach, treating each occurrence as separate, in order to prevent a substantial loss of cases from our data.
