# Supplementary material for: Bridging and bonding: The roles of brokerage and closure in mobilizing support provision in online support groups
Source: PLoS One. 2025 Jun 10;20(6):e0325108. doi: 10.1371/journal.pone.0325108 (PMC12151367; doi:10.1371/journal.pone.0325108)
Supplement: S2 Appendix — (DOCX) [file pone.0325108.s002.docx]

**Bridging and Bonding: The Roles of Brokerage and Closure in Mobilizing Support Provision in Online Support Groups**

**Supplemental Materials**

### **S2 Appendix. Social Support Provision: Manual Coding and Machine Leaning**

To categorize the provision of informational and emotional social support, we employed a supervised machine learning approach. To generate training and testing datasets for the machine learning model, five coders were enlisted. They evaluated the intended behavior of the content, determining whether the content was aimed at social support provision, social support seeking, or other purposes. If the content was deemed to be social support provision, coders further identified the type of support, discerning whether it was informational, emotional, or other types of support based on Cutrona & Russell’s classification [1]. Intercoder reliability was assessed using Krippendorff's alpha [2]. A substantial level of intercoder reliability was achieved over the training phase (α_intended behavior_ = .78; α_support type_ = .72) and the main task phase (α_intended behavior_ = .78; α_support type_ = .71). In total, the coders generated 7,654 labeled data entries. These labeled data were then partitioned into a training dataset (*n* = 6,123) and a testing dataset (*n* = 1,531) to enable machine learning-based classification.

For the automatic classification of intended behaviors (i.e., support provision; support seeking; others) and support types (i.e., information support; emotional support; others), this study utilized BERT, a natural language processing model initially developed by Google [3]. More specifically, we employed a Korean version of BERT (KoBERT), which was developed by a group of Korean researchers [4]. The pre-trained KoBERT was fine-tuned using our training dataset, with parameters set to 20 epochs and a batch size of 65. The performance of the fine-tuned KoBERT was evaluated using the testing dataset, based on the accuracy score, which denotes the proportion of cases correctly classified. The fine-tuned KoBERT achieved an accuracy score of 78% for intended behavior and 77% for support type. A message was designated as an information support provision if it was classified as support provision in intended behavior and information support in support type. Likewise, a message was classified as emotional support provision if it was identified as support provision in intended behavior and emotional support in support type.

**References**

1. Cutrona CE, Russell DW. Type of social support and specific stress: Toward a theory of optimal matching. 1990.

2. Krippendorff K. Content analysis: An introduction to its methodology. Sage publications; 2018.

3. Devlin J, Chang M-W, Lee K, Toutanova K. BERT: Pre-training of Deep Bidirectional Transformers for Language Understanding. arXiv; 2019. doi:10.48550/arXiv.1810.04805

4. SKT Brain. KoBERT. 2022. Available: https://github.com/SKTBrain/KoBERT#release
