# Supplementary material for: Bridging and bonding: The roles of brokerage and closure in mobilizing support provision in online support groups
Source: PLoS One. 2025 Jun 10;20(6):e0325108. doi: 10.1371/journal.pone.0325108 (PMC12151367; doi:10.1371/journal.pone.0325108)
Supplement: S3 Appendix — (DOCX) [file pone.0325108.s003.docx]

**Bridging and Bonding: The Roles of Brokerage and Closure in Mobilizing Support Provision in Online Support Groups**

**Supplemental Materials**

**S3 Appendix. Measurement of Information Support Quality**

The uniqueness of information between two texts can be quantified by assessing the degree of their semantic independence. To measure this uniqueness, we first estimated a semantic vector for each text using a Korean-sentence BERT-embedding model estimated based on KoBERT [1]. Text embedding is a technique in text mining that translates text into a numerical vector, facilitating a variety of text analyses, including semantic comparison. We chose BERT embedding due to its superior performance in document embedding compared to other models like TF-IDF embedding and doc2vec [2].

After estimating the vector for each text, we used a cosine similarity algorithm to measure the semantic similarity between the information provided by a focal individual and the information provided by another individual in a thread. Cosine similarity ranges from -1 (semantically opposite) to 1 (semantically identical). Values close to 0 indicate that two semantic vectors are independent, implying that the target information is semantically more unique compared to the other piece of information. Given that values close to 0 capture the uniqueness of information, we used the absolute value to ignore the sign of a cosine similarity score. Moreover, to make the measure more intuitive and pertinent to information uniqueness, we subtracted 1 from the absolute value of the cosine similarity. Consequently, a higher value indicates greater uniqueness of the target information compared to another piece of information, as the two texts are semantically distinct. For each thread where a focal person provided information support, the focal person's information support was compared with each piece of information support provided by others, then averaged. The uniqueness of the information provided by an individual ‘i’ is measured using the following formula:

Information uniqueness*_i_* = $\frac{\sum\frac{(1 -|cos \left( IV_{in}, {IV}_{jn} \right)|)}{n_{p}}}{n}$,

where IV_in_ represents the semantic vector of the focal person i’s information support provision to the nth post requesting support; IV_jn_ signifies the semantic vector of information support provided by another person (other than focal person i) to the nth post; n_p_ is the total number of pairs of focal person’s information support provisions that are compared with others’ support information provisions; and n is the total number of posts where the focal person i provides information support. A missing value was produced when no one, except for i, provided information support (*n* = 489) in a thread. In such instances, the missing value was replaced with the mean value of information uniqueness. Therefore, the information uniqueness of focal person i was estimated by dividing the sum of the average information uniqueness at the thread level by the number of threads in which the focal person provided information support.

**References**

1. jhgan00 (github user id). ko-sentence-transformers. 2021. Available: https://github.com/jhgan00/ko-sentence-transformers

2. Ajallouda L, Najmani K, Zellou A. Doc2Vec, SBERT, InferSent, and USE Which embedding technique for noun phrases? IEEE; 2022. pp. 1–5.
