# Supplementary material for: Bridging and bonding: The roles of brokerage and closure in mobilizing support provision in online support groups
Source: PLoS One. 2025 Jun 10;20(6):e0325108. doi: 10.1371/journal.pone.0325108 (PMC12151367; doi:10.1371/journal.pone.0325108)
Supplement: S4 Appendix — (DOCX) [file pone.0325108.s004.docx]

**Bridging and Bonding: The Roles of Brokerage and Closure in Mobilizing Support Provision in Online Support Groups**

**Supplemental Materials**

**S4 Appendix. Measurement of Emotional Support Quality**

The quality of emotional support provision is defined in this study as an emotional support message that encompasses both reflection and elaboration. Drawing from the person-centeredness framework, high-quality emotional support explicitly recognizes the support seeker and provides an extensive explanation of the support seeker’s feelings [1]. A high-quality emotional support provider is likely to accommodate their language to the seeker’s language to acknowledge and reflect the seeker’s feelings. This adaptation likely makes the support provision message semantically similar to the support-seeking post. This idea has been supported by previous research that utilized text similarity as an indicator of emotional support quality [2]. Furthermore, to elaborate on the recipient’s feelings, a high-quality emotional message will typically be more extensive than a lower quality message. Based on these theoretical considerations, this study computationally measures the quality of emotional support using a composite score. This score weights the text similarity between emotional support provision content and support seeking content (reflecting the degree of reflection) by the length of the emotional support provision content (indicating the degree of elaboration).

The Korean-sentence BERT-embedding model, which was used to measure information uniqueness, was also employed to estimate the semantic vectors of emotional support content. The following formula was used to represent the quality of emotional support of a focal person:

Quality of emotional support*_i_* = $\frac{\sum_{1}^{n} \cos\left( {EV}_{in}, {EV}_{n} \right)*{length}_{in}}{n}$ ,

where EV*_in_* represents the semantic vector of i’s emotional support provision to the nth post, and EV*_n_* is the semantic vector of the nth post. Meanwhile, length_in_ signifies the log-transformed and normalized length of i’s emotional support provision to the nth post. The length of i’s emotional support provision to the nth post was initially log-transformed and then normalized using the min-max normalization technique to align its scale with that of the cosine similarity. The min-max normalization technique fundamentally sets the minimum score to 0. This could potentially cause problems since it would result in a composite score of 0 when multiplying the cosine similarity score and length. To address this issue, a small constant score of .01 was added to the normalized score.

**References**

1. High AC, Dillard JP. A review and meta-analysis of person-centered messages and social support outcomes. Communication Studies. 2012;63: 99–118. doi:10.1080/10510974.2011.598208

2. Doré BP, Morris RR. Linguistic synchrony predicts the immediate and lasting impact of text-based emotional support. Psychol Sci. 2018;29: 1716–1723. doi:10.1177/0956797618779971
