# Supplementary material for: Bridging and bonding: The roles of brokerage and closure in mobilizing support provision in online support groups
Source: PLoS One. 2025 Jun 10;20(6):e0325108. doi: 10.1371/journal.pone.0325108 (PMC12151367; doi:10.1371/journal.pone.0325108)
Supplement: S5 Appendix — (DOCX) [file pone.0325108.s005.docx]

**Bridging and Bonding: The Roles of Brokerage and Closure in Mobilizing Support Provision in Online Support Groups**

**Supplemental Materials**

**S5 Appendix. Validity Assessment of Text Length as an Indicator of Emotional Support Elaboration**

In most person-centeredness studies, high quality messages are notably longer than low quality messages [1]. However, as the reviewer concerns, this does not guarantee that our data follow this general rule. To investigate whether our measurement was valid, we compared our approach with the large language model (LLM), which is a state-of-the-art technique. Previous studies demonstrated a high performance of LLMs in content analysis, showing that their performance outperforms other computational methods and is comparable to or sometimes surpasses human coders [2,3].

We employed GPT-4o, one of the most widely used LLMs, for our validation. Running the model through OpenAI's API with a temperature setting of 0.0, we ensured it generated consistent, predictable responses rather than creative variations that would be unnecessary for content analysis. We tasked GPT-4o with coding a subset of our support messages (*n* = 300) into three levels of elaboration (high, medium, or low) using the following prompt:

“*Your task is to analyze support messages and code them based on their level of elaboration (low, medium, or high). Message elaboration refers to the degree of detail, depth, and thoughtfulness in supportive communications. It encompasses how thoroughly a support provider addresses the specific concerns, emotions, or situations expressed by the support seeker. Elaboration includes personalization, contextual awareness, comprehensive addressing of concerns, detailed reasoning, and extended engagement beyond brief responses. Evaluate each support message and assign ONE of the following codes: low, medium, or high.*”

The correlation between our approach (i.e., text length) and LLM approach was 0.74. The high correlation coefficient between the two measures validates our use of text length as a proxy for message elaboration.

**References**

1. High AC, Dillard JP. A review and meta-analysis of person-centered messages and social support outcomes. Communication Studies. 2012;63: 99–118. doi:10.1080/10510974.2011.598208

2. Gilardi F, Alizadeh M, Kubli M. ChatGPT outperforms crowd workers for text-annotation tasks. Proc Natl Acad Sci USA. 2023;120: e2305016120. doi:10.1073/pnas.2305016120

3. Rathje S, Mirea D-M, Sucholutsky I, Marjieh R, Robertson CE, Van Bavel JJ. GPT is an effective tool for multilingual psychological text analysis. Proceedings of the National Academy of Sciences. 2024;121: e2308950121. doi:10.1073/pnas.2308950121
