# Supplementary material for: Bridging and bonding: The roles of brokerage and closure in mobilizing support provision in online support groups
Source: PLoS One. 2025 Jun 10;20(6):e0325108. doi: 10.1371/journal.pone.0325108 (PMC12151367; doi:10.1371/journal.pone.0325108)
Supplement: S6 Appendix — (DOCX) [file pone.0325108.s006.docx]

**Bridging and Bonding: The Roles of Brokerage and Closure in Mobilizing Support Provision in Online Support Groups**

**Supplemental Materials**

**S6 Appendix. Measurement of Non-redundant Information Environment**

To quantify the non-redundant information environment of a focal person, we first assembled the focal person’s information environment by collecting others’ messages that the focal person could have potentially seen. This study makes certain assumptions to pare down others’ messages to which a focal person may be exposed. When a focal person posts an update, we presuppose that they would read all comments, as all comments are directed at the focal person. When a focal person comments on a post authored by another person, it is assumed that the focal person has read the post and any comments made prior to their own.

After compiling the messages that a focal person is presumed to be exposed to, each message’s vector, estimated through the Korean-sentence BERT, was compared with one another, and the average semantic differences across messages were used to indicate the non-redundant information of a focal person. We established a threshold for the number of paired content, as it can be computationally exhaustive to compare each pair when a focal person’s information environment is extensive. If the number of paired contents is 1,000 or more, only 1,000 pairs were randomly selected and compared. The following formula was used to measure the non-redundant information environment of a focal person:

Non-redundant information environment*_i_* = $\frac{\sum(1-|cos \left( M_{j},M_{q} \right)|)}{n}$,

Let M_j_ and M_q_ are vectors of message j and message q, respectively. These messages were posted by others and presumably seen by the focal person, i. In this context, n represents the number of message pairs. The function measures the average semantic difference experienced by the focal person, i, across these message pairs.
