# Supplementary material for: Bridging and bonding: The roles of brokerage and closure in mobilizing support provision in online support groups
Source: PLoS One. 2025 Jun 10;20(6):e0325108. doi: 10.1371/journal.pone.0325108 (PMC12151367; doi:10.1371/journal.pone.0325108)
Supplement: S7 Appendix — (DOCX) [file pone.0325108.s007.docx]

**Bridging and Bonding: The Roles of Brokerage and Closure in Mobilizing Support Provision in Online Support Groups**

**Supplemental Materials**

**S7 Appendix. Measurement of Trust**

A composite index of self-disclosure and reciprocity was used to measure trust. An individual can be vulnerable to others by engaging in self-disclosure, which contains personal and sensitive information that could potentially jeopardizes the individual. Despite the potential risk, people engage in self-disclosure if they trust that others will not misuse the disclosed information. In that sense, self-disclosure serves as a proxy of one’s trust in others within the community [1]. Reciprocity is another behavior that manifests trust. Individuals engage in reciprocal interactions when they trust that others will reciprocate. In this sense, the more individuals exhibit reciprocal interactions the more they trust others in the community [2].

Self-disclosure was operationalized by counting the proportion of words related to self-disclosure including first-person singular pronouns (e.g., I, my), first-person plural pronouns (e.g., we, our), family (e.g., husband, mom), friends (e.g., neighbor, girlfriend), positive emotions (e.g., love), and negative emotions (e.g., sad, hurt) at the content level [3]. These values are then averaged at the user level to represent the self-disclosure of a focal person. A dictionary containing those self-disclosure words was created based on online materials [4,5] and the authors’ domain knowledge. To determine reciprocity, the proportion of reciprocal interactions for each user was calculated. For example, if a focal user A updated messages addressed to users B and C, and only user B responds to A, the reciprocal interaction for A is 0.5. Both self-disclosure and reciprocity measures were normalized, and a small constant value of 0.01 was added to both indices. This addition ensured that the multiplication would not yield zero when either of the values was zero. The product of these normalized and adjusted scores then represented the level of trust for the focal person.

**References**

1. Wheeless LR, Grotz J. The measurement of trust and its relationship to self-disclosure. Human Communication Research. 1977;3: 250–257. doi:10.1111/j.1468-2958.1977.tb00523.x

2. Adali S, Escriva R, Goldberg MK, Hayvanovych M, Magdon-Ismail M, Szymanski BK, et al. Measuring behavioral trust in social networks. 2010 IEEE International Conference on Intelligence and Security Informatics. Vancouver, BC, Canada: IEEE; 2010. pp. 150–152. doi:10.1109/ISI.2010.5484757

3. Huang K-Y, Chengalur-Smith I, Pinsonneault A. Sharing is caring: social support provision and companionship activities in healthcare virtual support communities. MISQ. 2019;43: 395–423. doi:10.25300/MISQ/2019/13225

4. Korean Family Terms. In: Wikipedia [Internet]. 23 Mar 2022. Available: https://ko.wikipedia.org/wiki/%ED%95%9C%EA%B5%AD%EC%9D%98_%EA%B7%BC%EC%B9%9C%EA%B0%84_%ED%98%B8%EC%B9%AD%EB%B2%95

5. On B-W, Park S, Na C. KunSentiLex. 2018. Available: https://github.com/park1200656/KnuSentiLex
